# Comparative Effect of Treadmill Exercise on Mature BDNF Production in Control versus Stroke Rats

**DOI:** 10.1371/journal.pone.0044218

**Published:** 2012-09-04

**Authors:** Aurore Quirié, Marie Hervieu, Philippe Garnier, Céline Demougeot, Claude Mossiat, Nathalie Bertrand, Alain Martin, Christine Marie, Anne Prigent-Tessier

**Affiliations:** 1 Unité INSERM U1093 Cognition, Action et Plasticité Sensorimotrice, Dijon, France; 2 Université de Bourgogne, Dijon, France; 3 Department of Neurology, University Hospital, Dijon, France; 4 EA 4267 Fonctions et Dysfonctions Epithéliales, Faculté de Médecine-Pharmacie, Besancon, France; University of Queensland, Australia

## Abstract

Physical exercise constitutes an innovative strategy to treat deficits associated with stroke through the promotion of BDNF-dependent neuroplasticity. However, there is no consensus on the optimal intensity/duration of exercise. In addition, whether previous stroke changes the effect of exercise on the brain is not known. Therefore, the present study compared the effects of a clinically-relevant form of exercise on cerebral BDNF levels and localization in control versus stroke rats. For this purpose, treadmill exercise (0.3 m/s, 30 min/day, for 7 consecutive days) was started in rats with a cortical ischemic stroke after complete maturation of the lesion or in control rats. Sedentary rats were run in parallel. Mature and proBDNF levels were measured on the day following the last boot of exercise using Western blotting analysis. Total BDNF levels were simultaneously measured using ELISA tests. As compared to the striatum and the hippocampus, the cortex was the most responsive region to exercise. In this region, exercise resulted in a comparable increase in the production of mature BDNF in intact and stroke rats but increased proBDNF levels only in intact rats. Importantly, levels of mature BDNF and synaptophysin were strongly correlated. These changes in BDNF metabolism coincided with the appearance of intense BDNF labeling in the endothelium of cortical vessels. Notably, ELISA tests failed to detect changes in BDNF forms. Our results suggest that control beings can be used to find conditions of exercise that will result in increased mBDNF levels in stroke beings. They also suggest cerebral endothelium as a potential source of BDNF after exercise and highlight the importance to specifically measure the mature form of BDNF to assess BDNF-dependent plasticity in relation with exercise.

## Introduction

Ischemic stroke is a leading cause of long-term motor disabilities. Other than tissue type plasminogen activator, there is no effective treatment for stroke and patients must rely on rehabilitation therapy to optimize recovery. Currently, there is increased emphasis on methods that intensify rehabilitation such as treadmill exercise with and without body support [1], [2], [3]. However, the biochemical mechanisms that underlie the benefits of exercise on the brain are still to be completely elucidated. It is clear that uncovering these mechanisms could lead to the optimization of exercise paradigms for the treatment of stroke. Animal research can directly examine the cellular and molecular cascades that are triggered by exercise.

Brain-Derived Neurotrophic Factor (BDNF) is central to many facets of adult brain function including synaptogenesis, neurogenesis, vasculogenesis and activity-dependent plasticity [4]. It is present in high amount in neurons of the central nervous system where it is initially synthesized as a precursor protein (pre-proBDNF) that is subsequently cleaved into proBDNF and mature BDNF (mBDNF). Once released, mBDNF activates TrkB receptors, thereby impacting positively brain function. BDNF has emerged as the main chemical translator of the neurophysiological effects of exercise on the intact brain [5], [6], [7], [8], [9], [10]. However, the crucial role of BDNF was identified from studies that have used free access to a running wheel during the animal’s dark cycle as a model of voluntary exercise. In contrast, the most popular form of exercise used in stroke patients is treadmill exercise, a form of forced exercise. As voluntary and forced exercises are not equivalent for their effect on brain and behavior [11], the possibility that these two forms of exercise may act through distinct pathways cannot be excluded. In addition, whether a given exercise paradigm impacts brain functioning through the same biochemical mechanisms in intact versus stroke brain has never been explored. Furthermore, despite increasing clinical evidence that task-repetitive training can induce adaptive neuroplasticity in the cortex [12], most studies on BDNF after exercise have focused on the hippocampus, a region which is however more involved in learning/memory than in motor function.

The present study was designed to investigate the regional effect of treadmill exercise on brain BDNF in intact and stroke brains. For this purpose, a 7 day-long treadmill walk (30 min/day) was induced in rats with or without ischemic stroke. Infarction was induced to the motor cortex by the photothrombotic ischemic stroke model that results in a lesion reproducible in volume and localization, and exercise was started after complete infarct maturation (on day 7 post-stroke). The day following the last boot of exercise, the levels of mBDNF and proBDNF in different brain regions including the lesion, the surrounding cortical areas, the hippocampus and the striatum were assessed using Western blotting analysis. ELISA tests were run in parallel to measure total BDNF proteins levels. Histological experiments were performed to investigate cellular BDNF localization and lesion volume. Synaptophysin was used as a marker of BDNF-dependent plasticity.

## Materials and Methods

### Animals

The experiments were carried out on adult male Wistar rats (300±10 g, Depré, Saint-Doulchard, France) that were housed five per standard cage at 21°C in a 12 h light/dark cycle (light on at 7 AM) with free access to food and water.

**Figure 1:**
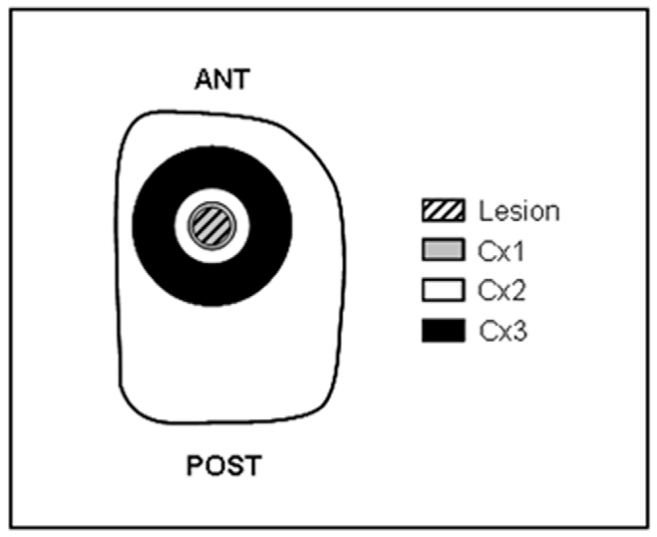
Cortical sampling. Three cortical areas (Cx1, Cx2 and Cx3 samples) were collected using concentric punches with increasing internal diameters (2.8, 4.6 and 9.5 mm), the first sample Cx1 being centered on the lesion in stroke rats or on the corresponding area in non stroke control rats. According to the dimensions of the lesion on day 15 post-stroke, Cx1 includes the entire lesion and a narrow surrounding perilesioned area while Cx2 and Cx3 correspond to rims more distant from the lesion. Cx = Cortex.

### Ethics Statement

The experimental procedures were conducted according to the French Department of Agriculture guidelines (license no. 21CAE035) and approved by the local committee for ethic in animal experimentation (Comité d’Ethique de l’Expérimentation Animale, Université de Bourgogne, 21079 Dijon, FRANCE). All surgery was performed under chloral hydrate anesthesia (400 mg/kg, i.p.) and all efforts were made to minimize suffering and stress of animals.

**Figure 2:**
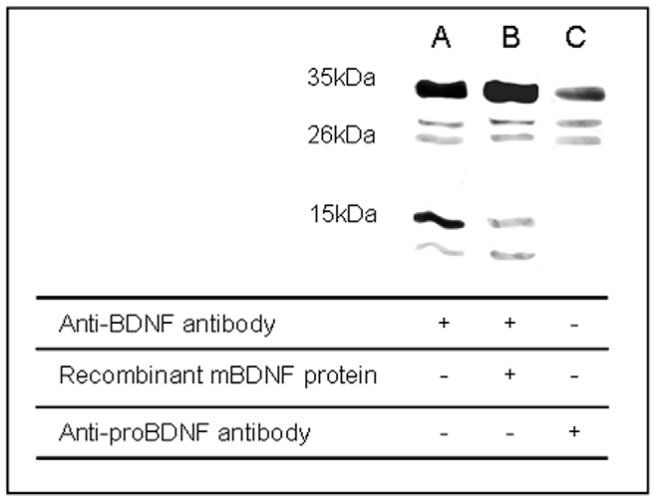
Specificity of antibodies used to measure mature BDNF and proBDNF by Western blotting analysis. Representative immunoblots from Cx3 samples collected in sedentary control. A) incubation of the membrane with anti-BDNF antibody, B) incubation of the membrane with anti-BDNF antibody in the presence of recombinant mature BDNF protein, C) incubation of the membrane with anti-proBDNF antibody.

**Figure 3:**
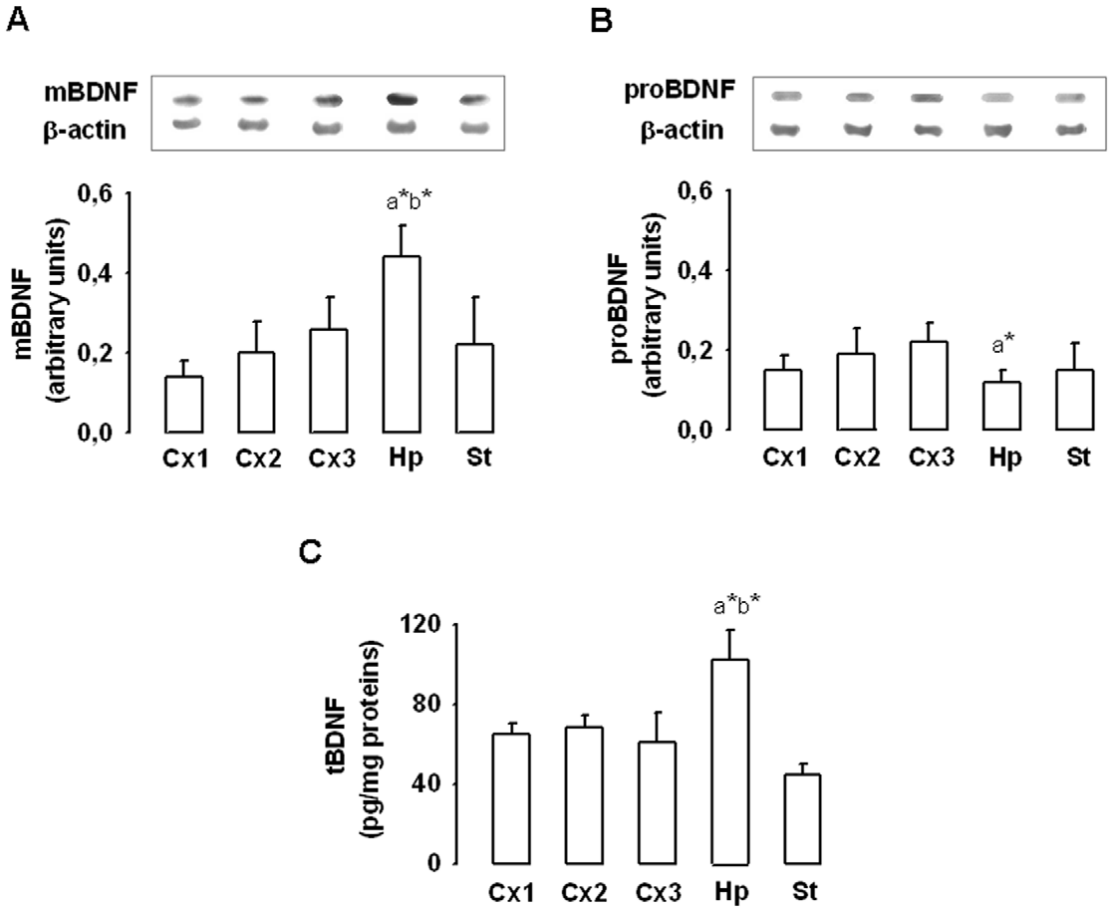
Regional distribution of mature BDNF (A), proBDNF (B) and total BDNF (C) levels in rat brain. Western blotting analysis was used to measure mature BDNF (mBDNF) and proBDNF whereas ELISA kit was used to assess total BDNF (tBDNF) in the cortex (Cx1, Cx2 and Cx3 regions), the hippocampus (Hp) and the striatum (St). Values normalized to β-actin are expressed as means ± SD from six sedentary control rats. a* different from the striatum, b* different from the cortical region with the highest levels (Cx 3 for A et B, Cx2 for C).

### Physical Exercise and Experimental Groups

First, all animals went through a five-day adaptation period to a rat treadmill (model Exer-3/6, Columbus Instruments) during which they were placed twice a day on a belt facing away from the electrified grid (0.6 mA intensity). The treadmill was turned on for few minutes at increasing speeds until reaching 0.3 m/s. This procedure has the purpose of excluding animals which failed to regularly run, providing a homogenous group of rats that will then be randomly allocated to one of the four groups: (1) sedentary control rats (SED control), (2) sedentary stroke rats (SED stroke), (3) exercised control rats (EX control), (4) exercised stroke rats (EX stroke). Exercised rats were trained 30 min/day, for 7 consecutive days at 0.3 m/s, a speed that is within the range of speed of rat’s overground locomotion [13]. According to [14], such a protocol corresponds to a mild aerobic exercise. The grid was not electrified during exercise. If necessary, a gentle touch on the hind limbs of the rats prevented them from drifting back on the treadmill. Exercise sessions were always performed between 9 and 12 AM. In stroke animals, exercise was started on day 7 after induction of photothrombosis.

**Figure 4:**
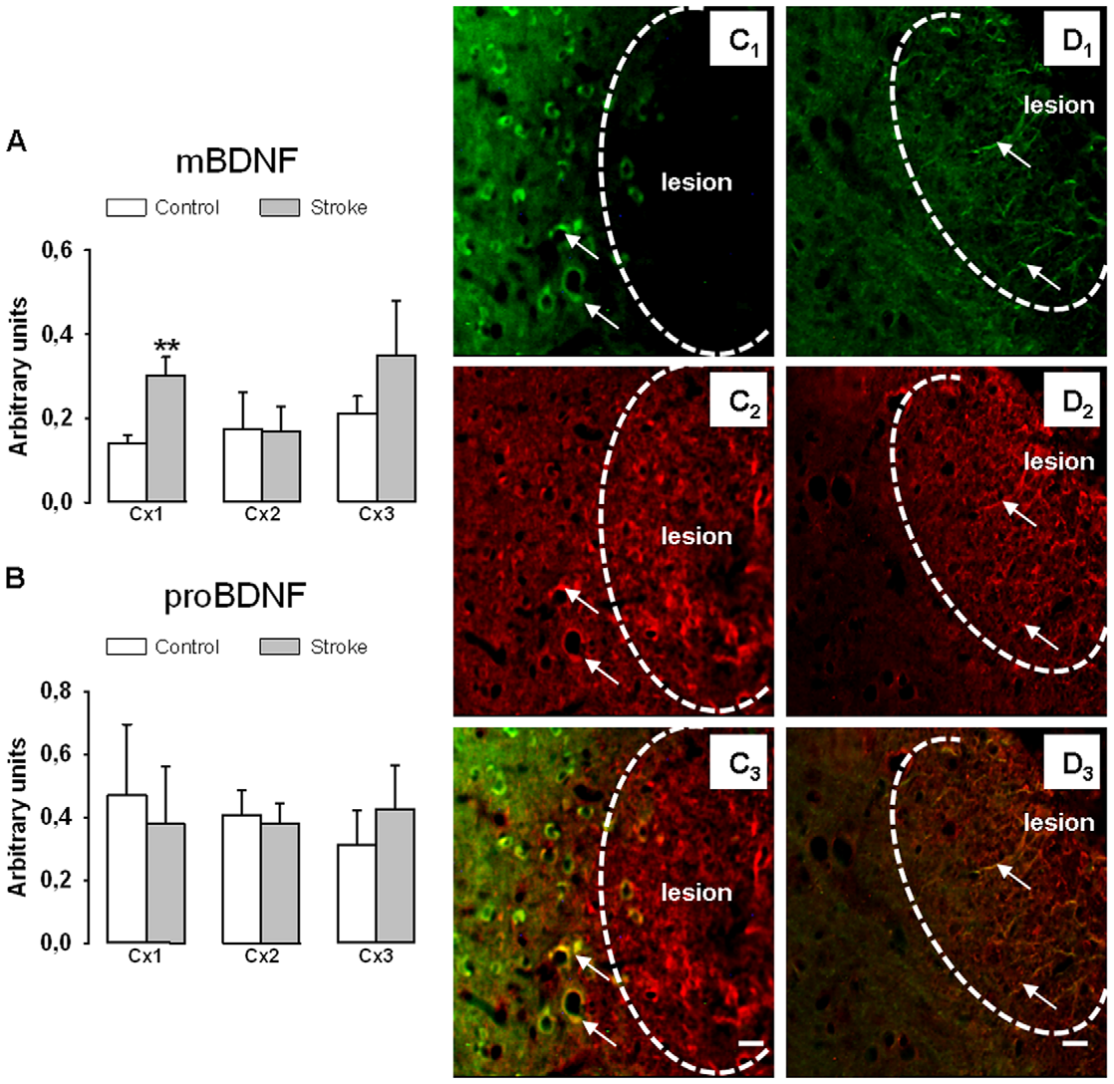
Effect of stroke on BDNF metabolism. Mature BDNF (A) and proBDNF(B) levels were measured in the cortical samples Cx1, Cx2 and Cx3. Values normalized to β-actin are expressed as means ± SD (n = 6 to 7 rats), **p<0.01 different from control rats. Representative photographs of brain sections passing through the lesion showing that BDNF was expressed by perilesional neurons as indicated by arrows (C1 =  NeuN labelling, C2 =  BDNF labelling, C3 =  overlay of BDNF with NeuN) and astrocytes at the lesion border (D1 =  GFAP labelling, D2 =  BDNF labelling, D3 =  overlay of BDNF with GFAP), as indicated by arrows. Scale bars = 20 µm.

**Figure 5:**
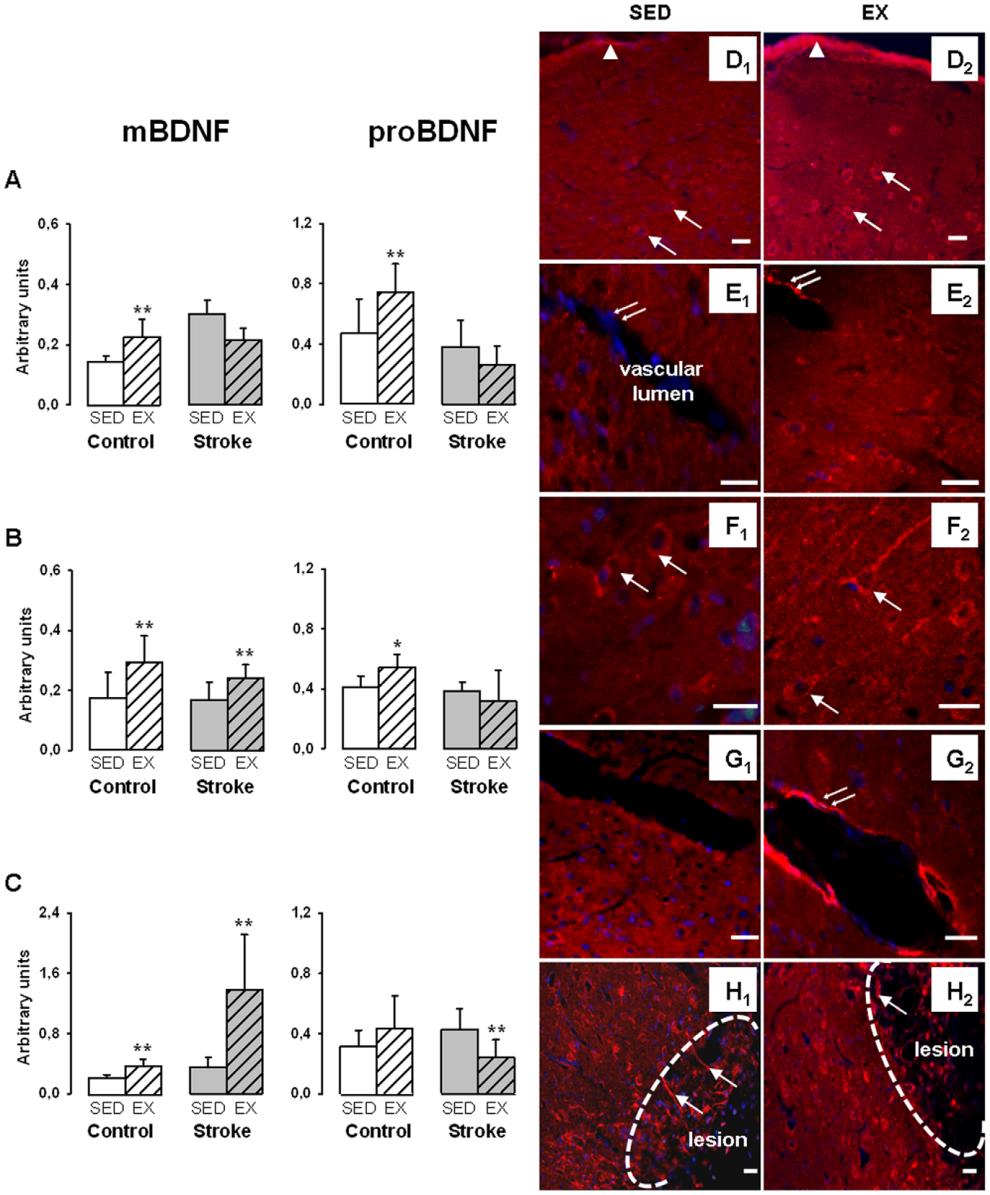
Comparative effect of exercise on cortical BDNF levels in control versus stroke rats. Mature BDNF (mBDNF) and proBDNF levels were assessed in sedentary (SED) or exercised (EX) rats in Cx1 (A), Cx2 (B) and Cx3 (C). Values normalized to β-actin are expressed as means ± SD (6 to 8 rats per group), *p<0.05, **p<0.01 different from sedentary rats. Representative photographs of brain sections showing BDNF localization in sedentary (SED) and exercised (EX) rats. DAPI (blue staining) was used to visualize nuclei. In control rats, exercise resulted in increased BDNF staining in neurons (arrows) and ependymal cells (arrow heads) of the pia (D1 vs D2) as well as in the appearance of BDNF staining in endothelial cells (double arrows) facing the vascular lumen of cerebral vessels (E1 vs E2). In stroke rats, neuronal BDNF staining (arrows) in regions distant from the lesion was stronger in EX than SED rats (F1 vs F2) and exercise resulted in the appearance of BDNF staining in endothelial cells (double arrows) of cerebral vessel endothelium (G1 vs G2). In contrast, we noticed that the number of astrocytes positive for BDNF (arrows) in the lesion vicinity was lower in EX stroke rats than SED stroke rats (H1 vs H2). Scale bars = 20 µm.

**Figure 6:**
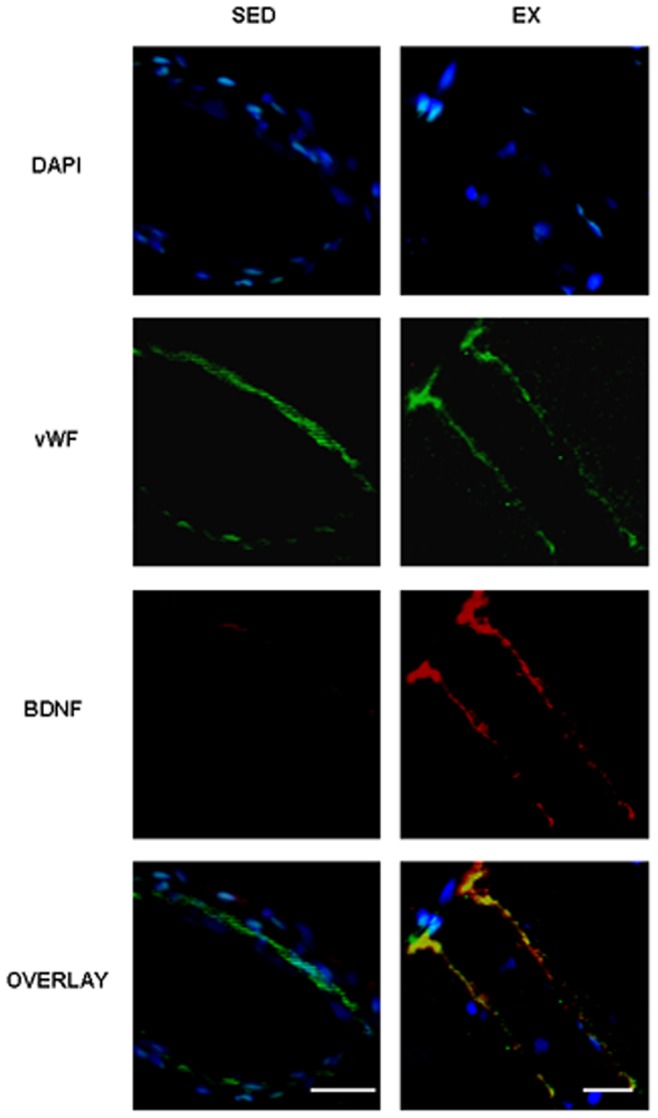
Endothelial BDNF localization after exercise. Representative photographs of brain sections collected from sedentary (SED) and exercised (EX) rats showing immunostaining for DAPI, vWF, BDNF and an overlay of the three fluorochromes. Note a co-localization of BDNF and vWF in EX rats only. Scale bars = 20 µm.

### Ischemic Stroke and Collection of Brain Samples

Infarction confined to the motor cortex was induced by the photothrombotic ischemic stroke model. Anesthetized rats were briefly placed on a stereotaxic apparatus. A laser beam was then focused with an optic fiber (1 mm internal diameter, emerging power 90 mW) on the skull according to the following coordinates: −0.5 mm AP and 3.5 mm right lateral relative to the bregma. The laser system was a diode-pumped solid-state laser (LCS-DLT-312, Opton Laser International, Orsay, France) working at 532 nm. The skull was irradiated for 6 min, the irradiation beginning 60 s before the infusion into the jugular vein of the photosensitizer dye rose bengal (20 mg/kg, 1 ml/kg for 20 s).

**Figure 7:**
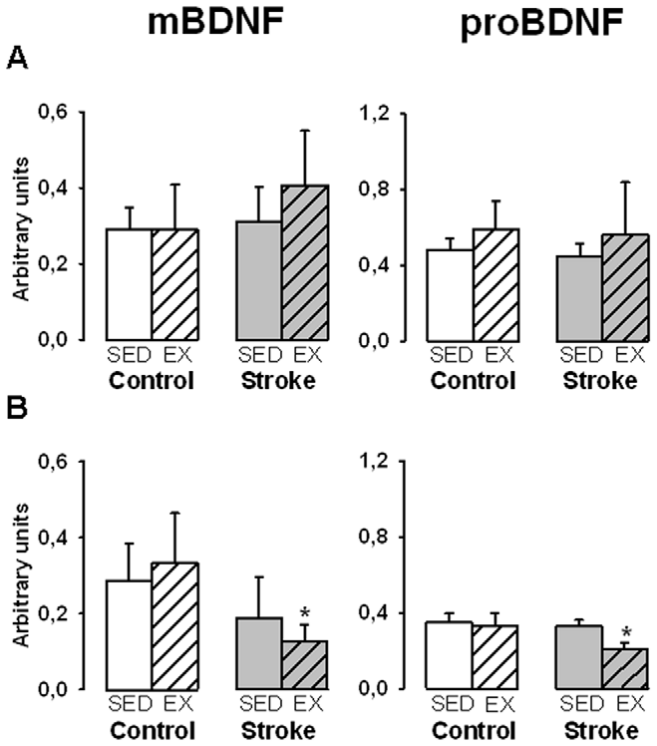
Comparative effect of exercise on subcortical BDNF levels in control versus stroke rats. Mature BDNF (mBDNF) and proBDNF levels were measured in sedentary (SED) or exercised (EX) rats in the hippocampus (A) and the striatum (B). Values normalized to β-actin are expressed as means ± SD (6 to 8 rats per group),*p<0.01 different from sedentary rats.

**Figure 8:**
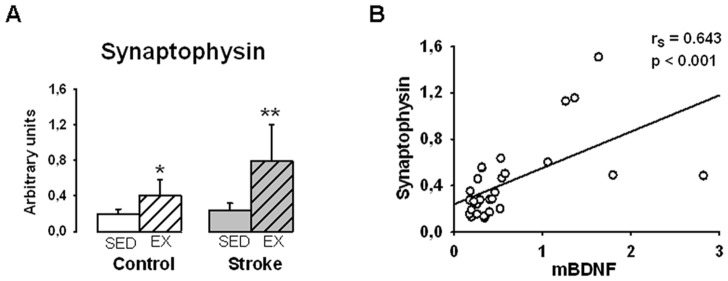
Relationship between synaptophysin and mature BDNF levels. Measurements were performed in sedentary (SED) and exercised (EX) control or stroke rats (n = 6 to 8 per group). A) Exercise increased synaptophysin expression in both control and stroke rats, *p<0.05, **p<0.01 different from sedentary rats. Values are expressed as means±SD. B) Synaptophysin and mBDNF levels were positively correlated. Each dot represents a rat. The measurements were performed in the cortex (Cx3 samples) and normalized to β-actin.

Brain sampling was carried out on day 15 after stroke induction and at corresponding time in control rats, i.e. ∼24 h after the last boot of exercise. After anesthesia and transcardiac perfusion with saline, brains were removed and dissected on ice. The cortex, the striatum and the hippocampus were collected from the hemisphere ipsilateral to the lesion. Three cortical areas (Cx1, Cx2 and Cx3) were sampled using concentric punches with increasing internal diameters (2.8, 4.6 and 9.5 mm), the first sample Cx1 being centered on the lesion in stroke rats or on the corresponding area in control rats. According to the dimensions of the lesion on day 15 post-stroke, Cx1 included the entire lesion and a narrow (less than 0.3 mm width) surrounding perilesioned area while Cx2 and Cx3 corresponded to rims more and less distant from the lesion as shown in Fig. 1. All the brain samples were immediately stored at −80°C until biochemical measurements.

### Histology

After anesthesia of animals, their brains were washed by transcardiac perfusion with saline and further perfused with 4% paraformaldehyde solution in 0.1 M phosphate buffer (pH 7.4). The brains were then removed, postfixed in the same fixative (1 h), transferred in a sucrose solution (20% in PBS for 48 h), frozen in isopentane (−40°C) and stored at −80°C. For determination of the infarct volume, coronal sections (20 µm thick) were cut at 200 µm interval in a cryostat (HM550, OMPV, Microm-Microtech, Francheville, France) at −20°C. The sections were collected on slides, and stained with Cresyl violet (0.4%, pH 3.5). Infarcted cortical areas, i.e. unstained tissue as a reflection of cell loss were measured using the computer image analysis system (Scion, NIH software, Bethesda, USA) and the distance between respective coronal sections were used to calculate a linear integration of the lesion volume. The lesion length (AP axis) was also measured. For BDNF localization, slices were collected on SuperFrost Plus slides. After rinsing in Tris Buffered Saline (TBS, pH 8.0), the sections were treated 10 min with proteinase K (10 µg/ml) at room temperature (RT) for antigen retrieval and incubated for 2 h in TBS containing 10% goat serum, 0.1% Triton X-100, 0.2% Tween 20 in order to block the non-specific binding sites. Thereafter, the sections were incubated for 3 days at 4°C in the same medium containing anti-BDNF antibody (Epitomics 3160-1, Euromedex, dilution 1/100) in the presence of antibodies recognizing either GFAP (G3893, Sigma-Aldrich, dilution 1/500), NeuN (MAB377, Merck-Millipore, dilution 1/500) or vWF (von Willebrand factor, MCA 127T, Sigma-Aldrich, dilution 1/100) in order to assess BDNF expression in astrocytes, neurons and endothelial cells respectively. After three washes for 10 min in TBS, antibody visualization was achieved by the incubation for 3 h (RT) with Alexa 488-conjugated anti-mouse (A11029) and/or Alexa 568-conjugated anti-rabbit IgGs (A11036, Interchim, Montluçon, France, dilution 1/1500). Negative controls were prepared by omitting the primary antibodies. Slices immunostained with BDNF alone or with both BDNF and vWF were then coverslipped with a fluorescent mounting containing 1.5 µg/ml of DAPI (Vectashield Mouting Medium with DAPI, Vector Laboratories,USA). Slices were observed with an epifluorescent microscope (Eclipse E600, Nikon). Experiments were performed in 5–10 successive brain sections in a same rat (n = 6 rats).

### Western Blotting Protocol

ProBDNF, mBDNF and synaptophysin expressions were determined by Western blotting analysis using anti-proBDNF (rabbit polyclonal, AB5613P, Merck-Millipore), anti-BDNF (rabbit monoclonal, Epitomics 3160-1, Euromedex) and anti-synaptophysin (rabbit polyclonal, Thermo scientific RB-1461-P1, Interchim), antibodies, respectively. The recombinant mature form of BDNF was purchased from Merck-Millipore (60237).

Brain samples were placed in 7 volumes of cold homogenization buffer (100 mM Tris, 150 mM NaCl, 1 mM EGTA, 1% triton X-100) to which a cocktail of protease inhibitors (P8340, Sigma-Aldrich) had been freshly added. Tissues were then homogenized, centrifuged (12,000 g, 20 min, +4°C) and the supernatants stored at −80°C until determination of the total protein concentrations by using the Lowry method. Equal volume of supernatant and Laemmli solution (62.5 mM Tris/HCl (pH 6.8), 2% SDS, 10% glycerol, 0.001% bromophenol blue) were mixed and 2-mercaptoethanol 5% was added. The mixture was heated at 85°C for 10 min. Proteins were then separated by SDS-PAGE on 12% polyacrylamide gels and transferred electrophoretically to a polyvinylidene difluoride (PVDF) membranes (0.2 µm, 200 mA, 2 h). After blocking non-specific binding sites with a 5% solution of non-fat dry milk in TBS (20 mM Tris/HCl, 137 mM NaCl, pH 7.4) containing 0.1% Tween 20, membranes were washed, incubated for 3 h (RT) with anti-proBDNF (1/2000), anti-BDNF (1/3000 with 5% non fat dry milk), anti-synaptophysin (1/2000) or anti-β-actin antibodies (1/5000, mouse monoclonal, A5441, Sigma-Aldrich) and then for 2 h with a horseradish peroxidase (HRP)-conjugated anti-mouse IgGs (1/50000, 115-035-166, Jackson ImmunoResearch) or anti-rabbit IgGs (1/30000, 1/50000, 1/80000 for mature BDNF, proBDNF and synaptophysin revelation, respectively, 115-035-144, Jackson ImmunoResearch). Protein-antibody complexes were visualized using the enhanced chemiluminescence Western blotting detection system according to the manufacturer’s protocol (ECL+, GE Healthcare, Orsay, France). β-actin was used as internal control.

Band densities were determined by scanning densitometry (GS-800, BIO-RAD, Ivry sur Seine, France). A computer-based imaging system (Quantity One, BIO-RAD, Ivry sur Seine, France) was used to measure the relative optical density of each band. Data were expressed as arbitrary units.

### ELISA Tests

Total BDNF (tBDNF) protein levels were determined with a commercial ELISA kit (ChemiKine™, Cat. No CYT306, MERCK MILLIPORE). According to the manufacturer, BDNF antibodies do not cross react with NGF, NT 4/5 or NT3 and do not discriminate the different forms of BDNF (mature BDNF, truncated proBDNF, proBDNF, preproBDNF). The limit of sensitivity was fixed at 6 pg/mL. The measurements were performed according to the manufacturer’s instructions. Supernatants of brain homogenates were diluted (1/10, v/v) in the homogenization buffer (see above for its composition). The diluted samples (50 µL) were again diluted (1/2, v/v) directly on the plate in a buffer provided by the kit. All assays were performed in duplicate. Brain tBDNF levels were expressed as pg/mg of proteins.

### Statistical Analysis

Values are expressed as means±standard deviation (SD). Statistical analysis was performed using non-parametric tests. The regional distribution of mature, pro- and total BDNF in the brain was analyzed using the Kruskall Wallis test followed by Mann-Whitney *U*-test and the Bonferroni’s procedure to reduce the risk I error. Using the test of Mann-Whitney *U*-test, we compared groups (1) and (2) to assess the effect of exercise in control rats, groups (3) and (4) to assess the effect of exercise in stroke rats, and groups (1) and (3) to assess the proper effect of stroke. The Spearman’s rank correlation coefficient (r_s_) was calculated to examine the dependence between mBDNF and synaptophysin levels. Statistical significance was set at P<0.05.

## Results

None of the rats were excluded from the study because of an unwillingness to walk on the treadmill and all stroke rats survived the different surgical procedures. In our experimental conditions, exercise did not modify the lesion volume. Indeed, infarct volume (that was measured at day 15 after stroke) was 5.40±1.96 mm^3^ in sedentary stroke rats (n = 6) and 5.43±1.98 mm^3^ (n = 6) in exercised stroke rats (data not shown). The lesion length was also not different between exercised (2.55±0.56 mm) and sedentary rats (2.37±0.37 mm).

### 1) Specificity of Antibodies used to Detect Different BDNF Forms

In studies showing immunoblots of BDNF, only intensity of special-interest bands are generally displayed, thus making difficult to get information of the specificity of the chosen antibodies. Here, specificity of antibodies directed towards BDNF and proBDNF was investigated on cortical brain tissues collected in sedentary control rats. The results are shown in Fig. 2. After incubation of the membrane with anti-BDNF antibody (Fig. 2A), five bands were observed below 50 kDa; a band at 35 kDa, two bands at around 26 kDa, a band at 15 kDa and a band at 11 kDa. The band at 15 kDa matched mBDNF as its intensity was specifically decreased when the membrane was incubated with anti-BDNF antibody in the presence of recombinant mBDNF protein (Fig. 2B). Incubation of the membrane with anti-proBDNF antibody (Fig. 2C) revealed a band at 35 kDa and two bands at approximately 26 kDa that corresponded to proBDNF and the two truncated forms of proBDNF, respectively. Preliminary experiments on brain tissue with anti-BDNF antibody ruled out the possibility to simultaneously measure proBDNF and mBDNF levels on the same blot due to important difference in their band intensities. Therefore, in the present study, proBDNF was measured using anti-proBDNF antibody (band at 35 kDa) and mBDNF using anti-BDNF antibody (band at 15 kDa).

### 2) Regional Distribution of mBDNF, proBDNF and tBDNF Levels in Controls

While the hippocampus is known as the cerebral region that contains the highest BDNF levels as measured with ELISA tests [15], [16], [17], studies that specifically examined the regional distribution of mBDNF and proBDNF levels in the brain are lacking. Our results revealed that the hippocampus contains the highest levels of mBDNF (Fig. 3A) but the lowest levels in proBDNF (Fig. 3B). They also confirmed that tBDNF are higher in the hippocampus than other regions (Fig. 3C).

### 3) Effect of Stroke, Exercise and Both on Mature BDNF and proBDNF Levels

Our data show that stroke alone (Fig. 4) changed BDNF metabolism selectively in Cx1 (the cortical region centered on the lesion). In this region, stroke increased mBDNF levels by a factor of two (Fig. 4A) but did not alter proBDNF levels (Fig. 4B). These effects coincided with a strong BDNF labelling of neurons (Fig. 4C1, 4C2, 4C3) and astrocytes (Fig. 4D1, 4D2, 4D3) present at the vicinity of the lesion. Notably, the closer to the lesion the neurons are, the more likely they exhibited strong BDNF labelling (see the gradient in neuronal BDNF staining, Fig. 4C3). The apparent differential BDNF staining in peri-lesional area was due to the fact that D2 image corresponded to capture of both the lesion and surrounding non lesioned area while C2 corresponded to capture of the lesion only. Under microscopic examination of the sections, BDNF staining in perilesional area was not different between C2 and D2 images. In other cortical regions Cx2 and Cx3, difference between control and stroke rats was observed neither for mBDNF (Fig. 4A) nor proBDNF (Fig. 4B) levels. Moreover, stroke did not modify mBDNF and proBDNF levels in the striatum as well as in the hippocampus (data not shown).

Fig. 5 compared the effect of exercise on the cortical levels of mBDNF and proBDNF in control versus stroke rats.

In control rats, exercise increased mBDNF and proBDNF levels in Cx1, Cx2 and Cx3 (Fig. 5A, B, C, respectively) even though the increase did not reach significance for proBDNF in Cx3. These changes were associated with increased BDNF labelling in neurons, ependymocytes of the pia (Fig. 5, D1 vs D2) as well as the appearance of a BDNF staining in endothelium of cerebral vessels present in the cortex (Fig. 5, E1 vs E2). Fig. 6 shows a colocalization of BDNF and vWF in EX but not SED rats.

In stroke rats, exercise did not significantly change mBDNF (−20%, NS) in Cx1 (Fig. 5A), but the levels were significantly increased in Cx2 (+43%, Fig. 5B) and Cx3 (+295%, Fig. 5C). With regard to proBDNF, exercise resulted in a significant decrease in the levels in Cx3 (−45%, p<0.05) and a trend towards a decrease in other cortical regions (−31% in Cx1 and −19% in Cx2, NS). Comparisons of BDNF immunostaining between sedentary and exercised stroke rats in cortical regions outside the lesion showed that exercise induced an increase in neuronal staining (Fig. 5, F1 vs F2) and the appearance of a strong BDNF staining of cerebral endothelium (Fig. 5, G1 vs G2). In the lesioned Cx1 region, a reduction of the number of BDNF positive astrocytes was noticed in response to exercise (Fig. 5, H1 vs H2) and may contribute to the reduction of mature and proBDNF levels observed in this region in exercised stroke rats as compared to sedentary stroke rats.

Fig. 7 compares the effect of exercise on the hippocampus and the striatum in control versus stroke rats. In the hippocampus (Fig. 7A), exercise was devoid of effect in control as well as in stroke rats. In contrast, while exercise had no effect in the striatum in control rats, it significantly reduced (−40%) the striatal content of the two BDNF forms in stroke rats (Fig. 7B).

### 4) Effect of Stroke, Exercise and Both on Total BDNF Levels

Irrespective of the considered region, ELISA-measured tBDNF levels were not significantly modified by stroke, exercise or both, as compared to respective control values (data not shown).

### 5) Synaptophysin Expression and Relationship between mBDNF and Synaptophysin Levels

In order to estimate the relationship between mBDNF tissue levels and cerebral plasticity, we plotted mBDNF levels against synaptophysin levels. The choice of synaptophysin as a marker of cerebral plasticity is based on its role on efficacy and number of synapses [18]. The measurements were performed in Cx3 collected in SED control, SED stroke, EX control and EX stroke. As shown in Fig. 8A, exercise resulted in a significant increase in synaptophysin expression both in control (+112%) and stroke rats (+238%). In addition, a significant positive correlation was observed between mBDNF and synaptophysin levels (r_s_ = 0.643, n = 28, p<0.001) (Fig. 8B).

## Discussion

Using a model of cortical ischemic stroke, the present study is the first to compare the effects of treadmill exercise on the levels of mature BDNF (mBDNF) and its precursor proBDNF in control versus stroke brain. Synaptophysin expression was used as a marker of mBDNF-dependent synaptic plasticity and photothrombosis of cortical vessels as a model of permanent cortical ischemic stroke. When induced in control rats, exercise resulted in upregulation of mBDNF and synaptophysin levels in the cortex. These effects of exercise were also observed in stroke rats. However, exercise induced opposite effects on proBDNF levels in control (elevation) and stroke (reduction) rats.

### Methodological Consideration

The use of Western blotting analysis to specifically measure mBDNF and/or proBDNF is rather recent. Until then, changes in BDNF metabolism were assessed from the measurements of BDNF mRNA (in situ hybridization and RT-PCR) or total protein (ELISA tests) levels. However, BDNF mRNA does not obligatory lead to the mature form of BDNF and ELISA tests do not allow distinguishing mBDNF from other forms of this neurotrophin. Consistently, the present study shows that ELISA-measured BDNF levels do not parallel mBDNF levels after exercise, highlighting the importance to specifically assess mBDNF in order to get a better understanding on the link between exercise and BDNF-dependent plasticity. Additionally, it has to be kept in mind that mBDNF levels found in tissue homogenates correspond to extra- and intra-cellular mBDNF, whereas only extracellular mBDNF can interact with TrkB receptors. Nevertheless, the present evidence of a positive correlation between mBDNF and synaptophysin levels (Fig. 7B) indicates that mBDNF levels found in tissue homogenates are predictive of its biological activity. Lastly, in the present study, exercise that was induced after complete lesion maturation did not change the lesion volume, thus excluding modulation of lesion volume as a confounding factor.

### Proper Effect of Stroke on BDNF

Most studies on BDNF after focal stroke were restricted to the measurement of BDNF at the lesion site. Consistent with a rapid induction of the transcription of BDNF gene in response to stroke, increased BDNF mRNA and proteins (ELISA tests) were observed in the first 24h after stroke onset as compared to preischemic values [19], [20], [21], [22], [23], [24]. Our results showed that mBDNF production occurred beyond the phase of infarct maturation and was still present during the phase of scar formation (15d post-stroke). At this delayed stage of stroke, intense BDNF staining was observed in neurons and astrocytes, suggesting that the two types of cells contribute to long-term adaptive changes that were reported in stroke animals and patients [25], [26], [27], [28], [29]. Notably, we previously reported a correlation between BDNF levels (ELISA tests) and GFAP expression (a marker of astrocytes activation) in stroke rats [30].

### Comparative Effect of Exercise on BDNF Metabolism in Control Versus Stroke Rats

The effect of exercise, either voluntary or forced, on the different BDNF forms is poorly documented. Recent studies reported increases in the mature form of BDNF but not of its precursor proBDNF in the hippocampus of rodents with free access to a running wheel as compared to their sedentary littermates [31], [32], [33], [34]. In contrast, our present results obtained in control rats reported a simultaneous increase in both forms of BDNF in response to treadmill exercise. In addition, the changes were confined to the cortex, sparing the hippocampus and the striatum. Notably, treadmill exercise in control rats was previously reported to increase mBDNF in the striatum [35], the spinal cord [36] but not in the hippocampus [37]. Taken together, these data are in contrast with the well-accepted idea that changes in BDNF metabolism induced by exercise are most apparent in the hippocampus and agree with the notion that voluntary and forced exercises are not equivalent for their effect on brain [11]. When induced in stroke rats, exercise was still able to stimulate mBDNF synthesis in the cortical regions distant from the lesion (Cx2 and Cx3). Importantly, the changes observed in stroke rats were of the same magnitude (for Cx2) or even higher (for Cx3) than those observed in control rats, indicating that the ischemic brain is equally and even more responsive to exercise than the intact brain. In control rats, it is accepted that increased mBDNF production mainly results from induction of gene encoding BDNF [10], [38]. Consistent with this mechanism, our results showed that exercise increased to a same extend proBDNF and mBDNF levels. A new finding of the present study is that mBDNF upregulation after exercise occurred without accompanied elevation in proBDNF levels in stroke rats, suggesting stimulation of the cleavage of proBDNF into mBDNF as a contributive mechanism to explain the effect of exercise in stroke rats. Many enzymes can be potentially involved such as convertases, furin, plasmin and matrix-metalloproteases. Although speculative, this hypothesis is compatible with the idea that stroke might have a priming effect on the cortex which in turn results in a more rapid production of mBDNF in response to exercise stimulus. Intriguingly, the content of the striatum in mBDNF and proBDNF levels decreased in response to exercise in stroke rats, while it was not different between sedentary and exercised control rats (Fig. 6). According to the connections between the cortex and the striatum and the reduced capacity of BDNF synthesis by striatal neurons [39], BDNF loss in the striatum after stroke may be linked to the stimulation or the inhibition by the lesion of BDNF axonal transport from the striatum towards the cortex or from the cortex to the striatum, respectively as BDNF can be transported in the axon in both anterograde and retrograde directions [39], [40]. Notably, the fact that exercise increased mBDNF in the cortex but not in subcortical regions supports the notion that the therapeutic efficacy of treadmill exercise after stroke [35], [41], [42], [43] is related to BDNF-dependent plasticity in this region. Consistently, it was proposed that the cortex of the damaged hemisphere may subserve motor recovery after stroke [27], [28]. Lastly, it is noteworthy that exercise was associated with the appearance of a strong BDNF staining in endothelium of cortical vessels. To the best of our knowledge, this is the first time that exercise is reported to affect cerebral BDNF metabolism in cells other than neurons. This finding may clearly open new avenues on the link between cardiovascular stimulation elicited by exercise and BDNF-dependent plasticity in the brain.

In conclusion, the present results are consistent with the involvement of BDNF-dependent plasticity in the beneficial effect of treadmill exercise after stroke. Although distinct biochemical mechanisms are likely to be involved in the production of mBDNF between exercised control and exercised stroke rats, it is important to highlight that exercise increases with a similar intensity mBDNF production in control and stroke animals. This finding suggests that control rats can be used to find optimal conditions of exercise (intensity, duration, modalities of contraction) that will result in increased mBDNF levels in stroke rats. The screening in control rats of the modalities of exercise would represent an important gain of time in the development of forced exercise as a therapeutic strategy in stroke. Future studies will aim to investigate the interaction of stroke combined to exercise on BDNF processing and to understand the mechanisms involved in BDNF overexpression by cerebral endothelium after exercise.
